# Towards Precision Medicine in Inflammatory Bowel Diseases: Pharmacogenetic Predictors of Ustekinumab Effectiveness and Persistence

**DOI:** 10.3390/ph19091428

**Published:** 2026-09-10

**Authors:** Ylenia Marino, Michelangelo Rottura, Claudia Ligresti, Antonio Battaglia, Clara De Francesco, Natasha Irrera, Vincenzo Arcoraci, Walter Fries, Anna Viola, Giovanni Pallio

**Affiliations:** 1 Department of Biomedical and Dental Sciences and Morphological and Functional Imaging, University of Messina, Via C. Valeria, 98125 Messina, Italy; ylenia.marino@unime.it (Y.M.); gpallio@unime.it (G.P.); 2 Department of Clinical and Experimental Medicine, University of Messina, Via C. Valeria, 98125 Messina, Italy; mrottura@unime.it (M.R.); ligresticlaudia@gmail.com (C.L.); battaglia.antonio.07@gmail.com (A.B.); clara904@hotmail.it (C.D.F.); nirrera@unime.it (N.I.); varcoraci@unime.it (V.A.); walter.fries@unime.it (W.F.); 3 Department of Health Promotion, Mother & Child Care, Internal Medicine & Medical Specialties, Gastroenterology & Hepatology Unit, University of Palermo, Piazza delle Cliniche, 90127 Palermo, Italy

**Keywords:** inflammatory bowel diseases, Crohn’s disease, ulcerative colitis, ustekinumab, single nucleotide polymorphisms, IL12B, rs6887695, precision medicine

## Abstract

**Background/Objectives**: Inflammatory bowel diseases (IBD), including Crohn’s disease (CD) and ulcerative colitis (UC), show substantial variability in response to biologic therapies. Ustekinumab, which targets the IL-12/23 pathway, is an established treatment for moderate-to-severe IBD; however, a proportion of patients fail to achieve sustained remission or discontinue treatment over time. This study aimed to evaluate the effectiveness and persistence of ustekinumab and to explore the potential role of pharmacogenetic markers in predicting treatment outcomes. **Methods**: In this observational cohort study, 98 IBD patients treated with ustekinumab, with or without corticosteroid bridge therapy, in routine clinical practice were enrolled. The primary endpoint was steroid-free remission (SFR) at 12 months, defined as clinical remission without concomitant corticosteroid use, whereas treatment discontinuation was evaluated during follow-up. Genomic DNA was extracted from peripheral blood samples, and four single-nucleotide polymorphisms (SNPs) in selected immune-related genes were analyzed: PTPN2 rs7234029, HLA-C rs10484554, IL23R rs11209026, and IL12B rs6887695. **Results**: Of the 98 patients included, 73 (74.5%) achieved SFR, whereas 25 (25.5%) did not. Carriers of IL12B rs6887695 variant genotypes showed higher odds of achieving SFR than wild-type patients after adjustment for disease type and baseline disease activity (OR = 4.77; 95% CI, 1.60–14.23; *p* = 0.005). During follow-up, 16 patients (16.3%) discontinued treatment. Carriers of IL12B rs6887695 variant genotypes also showed a significantly lower hazard of ustekinumab discontinuation than wild-type patients (HR = 0.29; 95% CI, 0.10–0.85; *p =* 0.024). **Conclusions**: This study provides real-world evidence supporting the effectiveness and persistence of ustekinumab in IBD patients. IL12B rs6887695 emerged as a potential pharmacogenetic marker associated with favorable ustekinumab outcomes; however, this exploratory and hypothesis-generating finding requires external validation in larger, independent cohorts before clinical application.

## 1. Introduction

Inflammatory bowel diseases (IBD), mainly Crohn’s disease (CD) and ulcerative colitis (UC), are chronic, relapsing inflammatory disorders of the gastrointestinal tract with a substantial worldwide impact. According to recent Global Burden of Disease 2021 analyses, approximately 3.83 million prevalent cases of IBD were estimated worldwide, confirming the epidemiological relevance of these diseases [1,2].

Although CD and UC differ in their anatomical distribution and histopathological features, both conditions are characterized by recurrent intestinal inflammation, abdominal pain, diarrhea, and a variable risk of complications and disease-related disability [3]. Specifically, UC is typically limited to the colonic mucosa, whereas CD may involve any segment of the gastrointestinal tract and is often characterized by transmural inflammation [4].

The pathogenesis of IBD is multifactorial and not fully understood. Current evidence suggests that genetic susceptibility, epithelial barrier dysfunction, alterations in the gut microbiota, environmental factors, oxidative stress, and dysregulated immune responses interact in promoting chronic intestinal inflammation [4,5].

Over the last decades, the therapeutic management of IBD has progressively shifted from symptom control toward treat-to-target strategies aimed at achieving sustained clinical remission, steroid-free remission (SFR), endoscopic improvement, and prevention of long-term complications [6,7]. Conventional therapies, including corticosteroids, immunomodulators, and aminosalicylates, remain useful in selected clinical settings, whereas biologic and small-molecule therapies have substantially expanded the therapeutic armamentarium. Anti-TNF agents have represented a major advance in IBD treatment and remain widely used; however, primary non-response, secondary loss of response, adverse events, and long-term safety concerns highlight the need for alternative therapeutic strategies and for biomarkers capable of improving treatment selection [8,9].

Among immune pathways involved in IBD, the IL-12/IL-23 axis has a central role in sustaining intestinal inflammation. IL-12 and IL-23 are heterodimeric cytokines that share the p40 subunit, encoded by the IL12B gene. IL-12 is composed of p35 and p40 subunits and promotes Th1-mediated immune responses, whereas IL-23 consists of p19 and p40 subunits and contributes to Th17 cell maintenance and expansion [10,11]. Dysregulation of this pathway has been implicated in both CD and UC, supporting its relevance as a therapeutic target [12].

Ustekinumab is a fully human IgG1 monoclonal antibody that binds the shared p40 subunit of IL-12 and IL-23, thereby inhibiting downstream signaling mediated by both cytokines. Its efficacy has been demonstrated in induction and maintenance therapy for moderate-to-severe CD and UC, establishing ustekinumab as an important therapeutic option in IBD patients, particularly in those with inadequate response or intolerance to conventional therapies or other biologics [13,14]. Long-term safety data and real-world evidence further support its role in the therapeutic management of IBD [15,16,17,18]. Nevertheless, not all patients achieve sustained remission or long-term treatment persistence, and interindividual variability in response remains a clinically relevant issue [19].

In this context, pharmacogenetics may help to identify genetic factors associated with treatment effectiveness and persistence. Several genes involved in immune regulation, IBD susceptibility, and biologic treatment response may contribute to interindividual variability in therapeutic outcomes [20,21,22]. Accordingly, the selected candidate genes reflect complementary aspects of immune-mediated intestinal inflammation and ustekinumab pharmacology. PTPN2 encodes a non-receptor protein tyrosine phosphatase involved in immune homeostasis, cytokine signaling, epithelial barrier regulation, and inflammatory responses, and has been investigated in relation to response to anti-IL-12/23 therapy in CD [23]. The HLA region contributes to antigen presentation and has been implicated in IBD susceptibility, particularly through MHC class II signals. In this context, HLA-C rs10484554 was selected because it has been investigated as an HLA-C/HLA-C*06:02-related variant in pharmacogenetic studies of biologic response, including anti-TNF and anti-IL-12/23 therapies [24,25]. Furthermore, IL23R encodes a receptor subunit involved in IL-23 signaling and represents one of the most consistently investigated loci in IBD genetics [26,27]. Within the same inflammatory axis, IL12B encodes the p40 subunit shared by IL-12 and IL-23, which represents the direct molecular target of ustekinumab. In addition, IL12B polymorphisms have been associated with IBD susceptibility and have also been investigated as potential predictors of ustekinumab response in immune-mediated diseases, making this gene of pharmacological and pharmacogenetic interest [28,29,30].

Based on this rationale, this study aimed to evaluate whether selected SNPs in PTPN2, HLA-C, IL23R, and IL12B genes may influence ustekinumab treatment effectiveness, assessed as SFR at 12 months, and treatment persistence, evaluated according to ustekinumab discontinuation during follow-up, in IBD patients. By addressing the pharmacogenetic determinants of these clinically relevant outcomes, this study may contribute to the development of more personalized and cost-effective therapeutic strategies in IBD.

## 2. Results

### 2.1. Patient Characteristics

A total of 98 patients were included in the study, with a median age of 54 years (IQR, 41–66); 46 patients (46.9%) were male. Seventy patients (71.4%) had CD, with a median baseline Harvey–Bradshaw Index (HBI) of 4.5 (IQR, 3.0–8.0), whereas 28 patients (28.6%) had UC, with a median baseline partial Mayo Score (pMS) of 4.5 (IQR, 2.0–6.0). Overall, 16 patients (16.3%) discontinued ustekinumab before completing the 12-month follow-up, whereas 82 patients (83.7%) remained on treatment for at least 12 months.

In the full cohort, 73 patients (74.5%) achieved SFR, whereas 25 (25.5%), including all patients who discontinued ustekinumab before month 12, were classified as non-SFR. No significant differences were observed between the SFR and non-SFR groups in age, sex, disease type, or baseline corticosteroid therapy. Among patients with CD, baseline disease activity was significantly higher in the non-SFR group, with a median HBI of 6.5 (IQR, 4–10), compared with 4 (IQR, 3–7) in the SFR group (*p* = 0.025). Similarly, among patients with UC, the median pMS was significantly higher in the non-SFR group than in the SFR group (6 [IQR, 5–7] vs. 3 [IQR, 1–6]; *p* = 0.019). No significant differences were observed between the SFR and non-SFR groups in the genotype distributions of PTPN2 rs7234029, HLA-C rs10484554, or IL23R rs11209026. Conversely, the genotype distribution of IL12B rs6887695 differed significantly between the two groups (*p* = 0.045), with the G/G genotype being more frequent among non-SFR patients than among SFR patients (60.0% vs. 32.9%; Table 1).

All patients were genotyped for the four selected SNPs: PTPN2 (rs7234029), HLA-C (rs10484554), IL23R (rs11209026), and IL12B (rs6887695). Genotype distributions were assessed for conformity with Hardy–Weinberg equilibrium (HWE), and all SNPs showed genotype distributions consistent with HWE in the study population (Table 2).

### 2.2. Association Between SNPs and Clinical Effectiveness

Patients with UC showed lower estimated odds of achieving SFR than patients with CD in the univariable analysis, although the association did not reach statistical significance (OR = 0.39; 95% CI, 0.15–1.01; *p* = 0.052). Moderate/severe baseline disease activity was significantly associated with lower odds of achieving SFR compared with clinical remission (OR = 0.13; 95% CI, 0.04–0.46; *p* = 0.001), whereas the association with mild disease activity was not statistically significant (OR = 0.35; 95% CI, 0.09–1.40; *p* = 0.138). Among the investigated polymorphisms, carriers of IL12B rs6887695 variant genotypes (heterozygous or homozygous) showed significantly higher odds of achieving SFR than wild-type patients in the univariable analysis (OR = 3.06; 95% CI, 1.20–7.82; *p* = 0.019).

In the multivariable model including disease type, baseline disease activity, and IL12B rs6887695, UC was not independently associated with SFR (OR = 0.49; 95% CI, 0.16–1.51; *p* = 0.212). Moderate/severe baseline disease activity remained independently associated with lower odds of achieving SFR (OR = 0.12; 95% CI, 0.03–0.46; *p* = 0.002), whereas the association with mild disease activity remained nonsignificant (OR = 0.33; 95% CI, 0.08–1.44; *p* = 0.142).

Carriers of IL12B rs6887695 variant genotypes showed significantly higher adjusted odds of achieving SFR than wild-type patients (OR = 4.77; 95% CI, 1.60–14.23; *p* = 0.005). No other clinical or genetic variables were significantly associated with SFR in univariable analyses (Table 3).

### 2.3. Association Between SNPs and Treatment Discontinuation

During the 12-month follow-up, 82 patients remained on ustekinumab therapy, whereas 16 patients discontinued treatment. Lack of benefit was the main reason for discontinuation (*n =* 13), while adverse events accounted for the remaining cases (*n =* 3; Supplementary Figure S1).

Significant differences between completers and discontinuers were observed in disease type and baseline disease activity. UC was more frequently represented among discontinuers than among completers (56.3% vs. 23.2%; *p* = 0.013), whereas CD was more frequent among completers (76.8% vs. 43.8%). Among patients with UC, discontinuers showed significantly higher baseline disease activity than completers, with a median pMS of 6 (IQR, 5–7) and 3 (IQR, 1–6), respectively (*p* = 0.046). Among patients with CD, the median HBI was numerically higher in discontinuers than in completers (8 [IQR, 3–10] vs. 4 [IQR, 3–7]), but the difference was not statistically significant (*p* = 0.278).

No significant differences were observed in age, sex, or baseline corticosteroid therapy. Furthermore, genotype distributions did not differ significantly between completers and discontinuers for any of the investigated SNPs (Table 4).

In univariable Cox regression analyses, UC was associated with a higher hazard of ustekinumab discontinuation than CD (HR = 3.67; 95% CI 1.36–9.86; *p* = 0.010). Moderate/severe baseline disease activity was also associated with a higher discontinuation hazard than clinical remission (HR = 5.12; 95% CI 1.43–18.36; *p* = 0.012). IL12B rs6887695 variant carriers showed a numerically lower discontinuation hazard than wild-type patients, although the association was not statistically significant (HR = 0.47; 95% CI 0.17–1.26; *p* = 0.132). No other clinical or genetic variable was significantly associated with treatment discontinuation in univariable analyses.

**Figure 1:**
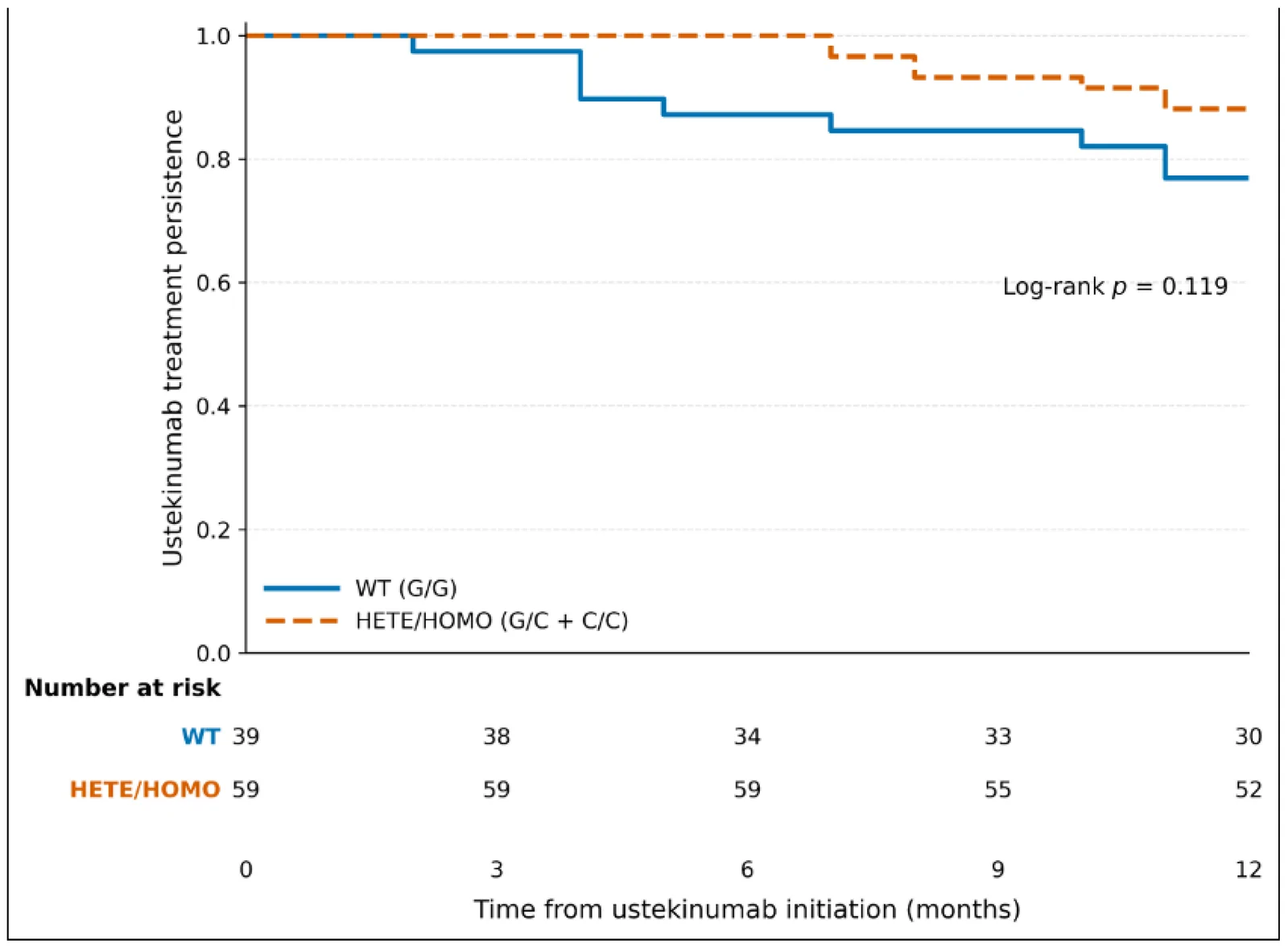
Kaplan–Meier curves of ustekinumab treatment persistence according to IL12B rs6887695 genotype. WT, wild type; HETE/HOMO, heterozygous or homozygous variant genotypes. Log-rank *p* = 0.119.

Given the limited number of discontinuation events, the multivariable Cox model was restricted to disease type, baseline disease activity, baseline corticosteroid therapy, and IL12B rs6887695. The reduced model showed a lower AIC than the full candidate model (135.25 vs. 139.81), while the addition of age, sex, PTPN2 rs7234029, HLA-C rs10484554, and IL23R rs11209026 did not significantly improve model fit (likelihood-ratio *p* = 0.365). In the final multivariable model, UC was associated with a higher hazard of treatment discontinuation than CD (HR = 3.81; 95% CI 1.26–11.55; *p* = 0.018), and moderate/severe baseline disease activity was associated with a higher hazard than clinical remission (HR = 4.66; 95% CI 1.20–18.09; *p* = 0.026). Mild disease activity and baseline corticosteroid therapy were not significantly associated with treatment discontinuation. IL12B rs6887695 variant carriers showed a lower discontinuation hazard than wild-type patients (HR = 0.29; 95% CI 0.10–0.85; *p* = 0.024; Table 5). No evidence of violation of the proportional-hazards assumption was observed for the final Cox model (global *p* = 0.523), and all covariate-specific tests were non-significant. Kaplan–Meier analysis showed numerically higher ustekinumab treatment persistence among carriers of IL12B rs6887695 variant genotypes than among wild-type patients; however, the difference did not reach statistical significance (log-rank *p* = 0.119; Figure 1).

## 3. Discussion

In this study, we evaluated whether selected polymorphisms in genes involved in immune-mediated inflammatory pathways may influence the clinical effectiveness and persistence of ustekinumab in patients with IBD. The main finding of this study was that IL12B rs6887695 remained associated with steroid-free remission after adjustment for disease type and baseline disease activity, whereas no significant univariable association with treatment effectiveness was observed for PTPN2 rs7234029, HLA-C rs10484554, or IL23R rs11209026. Similarly, carriers of IL12B rs6887695 variant genotypes showed a lower risk of ustekinumab discontinuation. Overall, these data point to IL12B rs6887695 as a potential determinant not only of clinical benefit, but also of the durability of ustekinumab treatment in IBD.

The association between IL12B rs6887695 and ustekinumab effectiveness is biologically plausible. IL12B encodes the p40 subunit shared by interleukin-12 and interleukin-23, which represents the direct molecular target of ustekinumab [31]. Therefore, genetic variation within IL12B may influence the inflammatory pathway on which ustekinumab acts and contribute to interindividual variability in response to p40 blockade. In our cohort, the wild-type genotype was more frequent among patients who did not achieve SFR, whereas heterozygous and homozygous variant genotypes were more represented among responders. Moreover, IL12B rs6887695 showed a favorable distribution of heterozygous and homozygous variant carriers in our cohort, allowing a reliable evaluation of the association between this SNP and clinical outcomes. These findings support the hypothesis that inherited differences in the IL-12/IL-23 pathway may identify a subgroup of IBD patients with greater probability of clinical benefit from ustekinumab.

Previous evidence directly linking IL12B rs6887695 to ustekinumab response is limited and mainly derives from psoriasis. Galluzzo et al. evaluated IL12B polymorphisms in patients with psoriasis treated with ustekinumab and reported that single SNP analysis did not show a statistically significant association between individual IL12B variants and treatment response [30]. By contrast, in our IBD cohort, IL12B rs6887695 remained associated with SFR after adjustment for disease type and baseline disease activity and was also associated with a lower risk of ustekinumab discontinuation. This observation is particularly relevant in intestinal immune-mediated inflammation and suggests that IL12B rs6887695 may represent a novel pharmacogenetic marker of ustekinumab effectiveness and persistence in IBD.

Additional support for the relevance of IL12B rs6887695 in IBD comes from genetic association studies evaluating disease susceptibility and phenotype. Glas et al. reported that IL12B rs6887695 modulates susceptibility and phenotype in IBD, although its effect appears less pronounced than that of IL23R variants [29]. Although disease susceptibility and pharmacological response are distinct outcomes, both may reflect altered IL12B-dependent immune signaling. Thus, our findings may extend the relevance of IL12B rs6887695 from IBD biology to ustekinumab pharmacogenetics.

The association between IL12B rs6887695 and treatment persistence further strengthens the clinical relevance of this variant. Treatment persistence is a clinically meaningful real-world outcome because it integrates effectiveness, tolerability, adherence, patient preference, and physician confidence in continuing therapy. In our cohort, treatment discontinuation was mainly driven by lack of benefit, whereas adverse events accounted for only a minority of cases. Therefore, the lower discontinuation risk observed among carriers of IL12B rs6887695 variant genotypes likely reflects more sustained disease control rather than a difference in safety. The concordance between SFR and Cox regression analyses suggests that IL12B rs6887695 may be a stronger candidate biomarker than variants associated with only one isolated endpoint.

In contrast, PTPN2 rs7234029 was not significantly associated with SFR or ustekinumab persistence in our cohort. This result deserves careful interpretation because Hoffmann et al. reported that the presence of at least one PTPN2 rs7234029 risk allele was associated with nonresponse to anti-interleukin-12/23 treatment in CD [23]. The biological plausibility of this locus is supported by previous evidence in immune-mediated diseases: Conigliaro et al. investigated PTPN2 rs7234029 in rheumatoid arthritis patients treated with TNF inhibitors and reported that this intronic variant may potentially modulate transcription factor binding sites involved in inflammatory pathways according to in silico analysis [32].

Our data do not confirm the association reported by Hoffmann et al. Several factors may explain this discrepancy. First, Hoffmann et al. evaluated only CD patients, whereas our cohort included both CD and UC. Second, although both studies considered clinically meaningful treatment failure, the outcome structure was not identical. Hoffmann et al. classified nonresponse within the first six months of anti-IL-12/23 therapy, whereas our analysis evaluated SFR and treatment persistence in an IBD cohort. Third, genotype distribution may have reduced the statistical power of our analysis. In our cohort, no homozygous variant carriers of PTPN2 rs7234029 were observed, whereas they are expected at low but measurable frequency in European populations. This difference does not invalidate the negative result, particularly because the variant was in Hardy–Weinberg equilibrium, but it may have limited our ability to detect modest genotype-response effects. Therefore, PTPN2 remains biologically and clinically interesting, but our findings suggest that rs7234029 alone may not be a consistent predictor of ustekinumab response across all IBD populations.

HLA-C rs10484554 was not associated with SFR or treatment persistence in our IBD cohort. The most direct comparison is provided by Masouri et al., who specifically evaluated rs10484554 in the HLA-C region, a SNP previously used as a proxy for the HLA-Cw6 locus. In that study, rs10484554 was associated with good response to anti-TNF agents, particularly adalimumab, whereas no clear association with ustekinumab response was observed [25]. This finding is consistent with our negative result and suggests that rs10484554 itself may not be a robust predictor of response to ustekinumab.

This result should be interpreted within the broader context of HLA-Cw6/HLA-C*06:02 pharmacogenetics in psoriasis. HLA-Cw6/HLA-C*06:02 is one of the most extensively investigated markers of ustekinumab response in psoriasis, with several studies showing an association with better clinical response and sustained PASI outcomes during follow-up. Talamonti et al. first reported that HLA-Cw6 predisposed to clinical response to ustekinumab in psoriasis [33] and subsequent studies in Caucasian and European real-life cohorts confirmed its association with better clinical response, including sustained PASI responses during long-term follow-up [34,35]. Galluzzo et al. also confirmed the positive role of HLA-Cw6 as a predictor of response to ustekinumab in psoriasis [30]. In a large psoriasis cohort, Dand et al. further showed that HLA-C*06:02 stratifies differential response to adalimumab and ustekinumab, supporting the concept that HLA-C*06:02-positive and HLA-C*06:02-negative psoriasis may represent biologically distinct treatment-response endotypes [36].

Therefore, although HLA-Cw6/HLA-C*06:02 has a well-established role in psoriasis pharmacogenetics, the absence of association between HLA-C rs10484554 and ustekinumab outcomes in our cohort suggests that HLA-C-related pharmacogenetic effects are likely disease-specific. HLA-Cw6/HLA-C*06:02 is strongly linked to psoriasis susceptibility and antigen presentation in cutaneous inflammation, whereas it is not a central determinant of IBD pathogenesis. Accordingly, the pharmacogenetic relevance of HLA-C variation in psoriasis should not be directly extrapolated to intestinal immune-mediated inflammation.

IL23R rs11209026 was not associated with ustekinumab effectiveness or persistence in our study. This variant is one of the most extensively studied protective alleles in IBD susceptibility. Bank et al. reported that IL23R rs11209026 was associated with reduced risk of CD and IBD in a Danish cohort, supporting the relevance of the IL-23 pathway in intestinal inflammation [37]. More recent mechanistic evidence confirms that IL-23 plays a central role in the differentiation and maintenance of Th17 cells and that IL23R R381Q/rs11209026 is linked to reduced risk of both CD and UC, probably through attenuation of downstream inflammatory signaling, including STAT3/STAT4 activation and Th17-related cytokine production [27]. Our negative finding is consistent with previous IBD pharmacogenetic evidence. Hoffmann et al. did not find IL23R rs11209026 to be associated with response to anti-IL-12/23 treatment in CD [23]. Thus, although IL23R rs11209026 remains important for understanding IBD susceptibility and IL-23 pathway biology, available evidence does not support its role as a robust predictive biomarker of ustekinumab effectiveness or persistence. This may be explained, at least in part, by the relatively low frequency of this variant and by the fact that ustekinumab targets the shared p40 subunit of IL-12 and IL-23 rather than the IL-23 receptor itself.

From a clinical perspective, our findings suggest that IL12B rs6887695 may represent a promising candidate biomarker for ustekinumab treatment selection in IBD. Its potential value is supported by three elements: the gene encodes the direct molecular target of ustekinumab; the association remained significant after adjustment for disease type and baseline disease activity; and the same variant was associated with both SFR and lower treatment discontinuation risk. Nevertheless, this marker should currently be considered hypothesis-generating rather than ready for clinical implementation. Recent systematic reviews of pharmacogenetic studies in IBD have emphasized that available evidence remains heterogeneous, with differences in study design, population, ethnicity, biological agent, endpoint definition, and follow-up duration [38,39]. In addition, most pharmacogenetic data in IBD still concern anti-TNF agents, whereas evidence for ustekinumab and vedolizumab remains comparatively limited [38,39]. A realistic future strategy may therefore involve integrating IL12B rs6887695 with inflammatory biomarkers, disease phenotype, and possibly multi-locus genetic scores.

This study has several strengths. First, we focused on biologically plausible variants in genes involved in pathways relevant to ustekinumab mechanism of action or immune-mediated inflammatory disease. Second, all genotype distributions were compatible with HWE. Third, we evaluated both SFR and treatment persistence, allowing us to assess not only clinical effectiveness but also durability of benefit in real-world practice. Fourth, the inclusion of both CD and UC reflects current clinical use of ustekinumab across the IBD spectrum. Finally, the concordant association of IL12B rs6887695 with both effectiveness and persistence strengthens the internal consistency of the finding.

### Limitations and Future Perspectives

Several limitations should also be acknowledged. The sample size was modest, and the limited number of non-SFR outcomes and treatment discontinuation events may have reduced the precision and stability of the multivariable estimates, as reflected by the wide confidence intervals. Despite restricting the Cox model to clinically relevant covariates and IL12B rs6887695, the low events-per-parameter ratio means that residual model overfitting cannot be excluded. Some variants also had a low minor allele frequency, and endoscopic endpoints or mucosal healing were not evaluated.

An additional limitation is that therapeutic drug monitoring (TDM), including the measurement of serum ustekinumab concentrations and anti-drug antibodies, was not performed. Consequently, the relationship between IL12B rs6887695, ustekinumab exposure, and clinical outcomes could not be evaluated. Future prospective studies should assess the implementation of TDM alongside IL12B rs6887695 genotyping to determine whether this combined approach may improve the prediction of treatment effectiveness and persistence and support treatment optimization.

Furthermore, the absence of a formal correction for multiple comparisons may increase the risk of false-positive findings; therefore, the genetic associations should be interpreted as exploratory and hypothesis-generating.

Finally, external validation in larger, independent, prospectively followed IBD cohorts is required before IL12B rs6887695 can be considered for clinical decision-making.

## 4. Materials and Methods

### 4.1. Patient Recruitment and Data Collection

A single-center observational pharmacogenetic study was carried out at the IBD Unit of the “G. Martino” University Hospital of Messina. The study protocol was approved by the Ethics Committee of Messina (identification number 69-25, 1 July 2025). Following protocol approval, patients with a confirmed diagnosis of CD or UC who were treated with ustekinumab were recruited after receiving detailed information about the study and providing written informed consent.

The final study population included 98 patients treated with ustekinumab. Therapeutic outcomes were evaluated during the first 12 months after treatment initiation or up to the time of treatment discontinuation when this occurred before month 12. At enrollment, peripheral blood was collected for genetic analysis. Demographic and clinical information was retrieved from medical records and follow-up documentation.

Patient care and treatment monitoring were conducted according to standard clinical practice. The following variables were collected: sex, age, IBD diagnosis, date of ustekinumab initiation, disease activity, concomitant corticosteroid therapy, treatment discontinuation, and reasons for discontinuation. Disease activity was assessed using the HBI in patients with CD and pMS in patients with UC [40,41].

Because the HBI and pMS are disease-specific instruments with different numerical ranges, their raw scores are not directly comparable in analyses including both CD and UC. Therefore, baseline disease activity was harmonized by mapping each score to four corresponding clinically defined categories of increasing activity, allowing disease activity to be included as a common categorical covariate for both diseases while preserving the disease-specific interpretation of each instrument. Patients were classified as being in remission, or having mild, moderate, or severe disease activity according to the established HBI and pMS cut-off values (HBI: remission <5, mild 5–7, moderate 8–16, severe >16; pMS: remission <2, mild 2–4, moderate 5–7, severe >7) [42]. For regression analyses, the moderate and severe categories were combined because of the limited number of patients with severe disease activity, yielding a three-level categorical variable comprising clinical remission, mild activity, and moderate/severe activity, with clinical remission serving as the reference category in both logistic and Cox regression models.

SFR was evaluated in the full cohort at 12 months. Patients remaining on ustekinumab treatment at month 12 were classified as achieving SFR if they were in clinical remission (HBI < 5 for CD or pMS < 2 for UC) without concomitant corticosteroid therapy. Patients remaining on treatment who did not meet these criteria, together with all patients who discontinued ustekinumab before month 12, were classified as non-SFR.

### 4.2. SNP Selection, DNA Extraction, and Genotyping

SNPs were selected according to a candidate-gene strategy, focusing on variants with biological plausibility or previous genetic/pharmacogenetic evidence in immune-mediated inflammatory diseases and pathways potentially involved in ustekinumab pharmacodynamics. Particular attention was given to genes related to the IL-12/IL-23 axis, immune regulation, and HLA-mediated antigen presentation. Based on these criteria, four SNPs were analyzed: IL12B rs6887695, IL23R rs11209026, PTPN2 rs7234029, and HLA-C rs10484554.

Blood samples for genetic analysis were obtained by venipuncture using 3-mL ethylenediaminetetraacetic acid (EDTA)-containing tubes. Genomic DNA was isolated from whole blood using the QIAamp DNA Blood Mini Kit (Qiagen, Hilden, Germany), following the manufacturer’s protocol. After extraction, DNA was eluted in the provided elution buffer, quantified spectrophotometrically, and stored at −20 °C until genotyping.

Genotyping was performed using predesigned TaqMan SNP Genotyping Assays (Thermo Fisher Scientific, Waltham, MA, USA). Assays were prepared as 20× working solutions in 1× Tris–EDTA (TE) buffer (10 mM Tris-HCl, 1 mM EDTA, pH 8.0, sterile DNase-free filtered water), according to the manufacturer’s recommendations. PCR reactions were carried out in 96-well MicroAmp™ optical reaction plates in a final volume of 25 μL per well, consisting of 12.50 μL of 2× TaqMan Master Mix, 1.25 μL of 20× assay mix, 1 μL of genomic DNA at 10 ng/μL, and 10.25 μL of nuclease-free water. Plates were sealed and briefly centrifuged before amplification.

Real-time PCR was conducted on the QuantStudio 6 Flex Real-Time PCR System (Applied Biosystems, Foster City, CA, USA). The thermal cycling protocol consisted of an initial denaturation step at 95 °C for 10 min, followed by 40 cycles at 95 °C for 15 s and 60 °C for 1 min [43,44,45,46]. Genotypes were assigned by allelic discrimination analysis using the instrument software and independently reviewed by two investigators. Discordant calls, if any, were resolved by consensus.

### 4.3. Data Analysis

A descriptive analysis of demographic and clinical characteristics was performed. Continuous variables were reported as median and interquartile range (IQR; Q1–Q3), whereas categorical variables were expressed as absolute frequencies and percentages. Patients were stratified according to treatment effectiveness into SFR and non-SFR groups, and according to treatment persistence into completers and discontinuers.

The distribution of continuous variables was assessed using the Kolmogorov–Smirnov test. Since the variables did not follow a normal distribution, non-parametric analyses were applied. Continuous variables were compared using the Mann–Whitney U test, whereas categorical variables were analyzed using Fisher’s exact test, including its extension to 2 × 3 contingency tables when appropriate. Genotype and allele frequencies were calculated for each investigated SNP, and HWE was assessed using the exact test. For regression analyses, patients carrying at least one variant allele, including heterozygous and homozygous variant genotypes, were grouped and compared with patients homozygous for the major allele.

Univariable logistic regression models were used to evaluate clinical and genetic factors associated with SFR at 12 months. Variables showing an association with SFR at *p* < 0.10 in univariable analyses were subsequently included in a single parsimonious multivariable logistic regression model. The final multivariable model included disease type, baseline disease activity and IL12B rs6887695. Results were reported as odds ratios (ORs) with corresponding 95% confidence intervals (95% CIs).

Kaplan–Meier curves were compared using the log-rank test. Time-to-treatment discontinuation was analyzed using univariable and multivariable Cox proportional hazards regression models. Given the limited number of discontinuation events, the multivariable model was restricted to disease type, baseline disease activity, baseline corticosteroid therapy, and IL12B rs6887695. Age, sex, and the remaining candidate SNPs were excluded from the reduced multivariable model to preserve parsimony. The reduced model was compared with the original model, which included all evaluated clinical and genetic variables, using a likelihood-ratio test and the Akaike information criterion (AIC). The proportional-hazards assumption was formally assessed by testing covariate-by-log (time) interaction terms. Hazard ratios (HRs) with corresponding 95% CIs were calculated to estimate the association between clinical and genetic variables and the risk of ustekinumab discontinuation during follow-up.

The candidate SNPs and study outcomes were defined a priori. Given the exploratory and hypothesis-generating nature of the study, no formal correction for multiple comparisons was applied.

A two-sided *p*-value < 0.05 was considered statistically significant. All statistical analyses were performed using SPSS Statistics version 23.0 (IBM Corp., Armonk, NY, USA).

## 5. Conclusions

In conclusion, this study identifies IL12B rs6887695 as a potential pharmacogenetic marker of ustekinumab effectiveness and persistence in IBD. Carriers of IL12B rs6887695 variant genotypes showed a higher probability of achieving SFR and a lower hazard of treatment discontinuation. These findings reinforce the concept that genetic variation within the IL-12/IL-23 pathway may contribute to the heterogeneity of ustekinumab response and support further investigation of IL12B rs6887695 as part of a broader precision-medicine strategy in IBD.

## Figures and Tables

**Table 1:** Patients’ characteristics and genotype distributions of selected SNPs stratified according to steroid-free remission status (SFR vs. non-SFR).

Variable	Total	SFR	Non-SFR	*p*-Value
	98	73	25	
Age [median, Q1–Q3]	54 (41–66)	55 (40–66)	50 (42–63)	0.636
Gender (M)	46 (46.9)	35 (47.9)	11 (44.0)	0.818
Disease				
CD	70 (71.4)	56 (76.7)	14 (56.0)	0.071
UC	28 (28.6)	17 (23.3)	11 (44.0)	
Baseline disease activity				
HBI [median, Q1–Q3]	4.5 (3–8)	4 (3–7)	6.5 (4–10)	0.025
pMS [median, Q1–Q3]	4.5 (2–6)	3 (1–6)	6 (5–7)	0.019
Baseline steroid therapy	27 (27.6)	18 (24.7)	9 (36.0)	0.305
PTPN2 rs7234029				
A/A	74 (75.5)	53 (72.6)	21 (84.0)	0.295
A/G	24 (24.5)	20 (27.4)	4 (16.0)	
G/G	0 (0.0)	0 (0.0)	0 (0.0)	
HLA-C rs10484554				
C/C	72 (73.5)	52 (71.2)	20 (80.0)	0.829
C/T	21 (21.4)	17 (23.3)	4 (16.0)	
T/T	5 (5.1)	4 (5.5)	1 (4.0)	
IL23R rs11209026				
G/G	89 (90.8)	66 (90.4)	23 (92.0)	0.320
G/A	8 (8.2)	7 (9.6)	1 (4.0)	
A/A	1 (1.0)	0 (0.0)	1 (4.0)	
IL12B rs6887695				
G/G	39 (39.8)	24 (32.9)	15 (60.0)	0.045
G/C	39 (39.8)	31 (42.5)	8 (32.0)	
C/C	20 (20.4)	18 (24.7)	2 (8.0)	

**Table 2:** Hardy–Weinberg equilibrium analysis of the SNPs included in the study.

Gene	SNP	Genotype Distribution			Observed Heterozygosity	Expected Heterozygosity	HWE
PTPN2	rs7234029	A/A	A/G	G/G	24.5%	21.5%	0.349
		74 (75.5%)	24 (24.5%)	0 (0.0%)			
HLA-C	rs10484554	C/C	C/T	T/T	21.4%	26.6%	0.059
		72 (73.5%)	21 (21.4%)	5 (5.1%)			
IL23R	rs11209026	G/G	G/A	A/A	8.2%	9.7%	0.214
		89 (90.8%)	8 (8.2%)	1 (1.0%)			
IL12B	rs6887695	G/G	G/C	C/C	39.8%	48.1%	0.094
		39 (39.8%)	39 (39.8%)	20 (20.4%)			

**Table 3:** Univariable and multivariable logistic regression analyses of factors associated with steroid-free remission at 12 months.

Variable	Univariable OR (95% CI)	*p*-Value	Multivariable OR (95% CI)	*p*-Value
Age	1.01 (0.98–1.04)	0.715	–	–
Gender (M)	1.17 (0.47–2.92)	0.733	–	–
Disease				
CD	Reference		Reference	
UC	0.39 (0.15–1.01)	0.052	0.49 (0.16–1.51)	0.212
Baseline disease activity				
Clinical remission	Reference		Reference	
Mild activity	0.35 (0.09–1.40)	0.138	0.33 (0.08–1.44)	0.142
Moderate/severe activity	0.13 (0.04–0.46)	0.001	0.12 (0.03–0.46)	0.002
Baseline steroid therapy	0.58 (0.22–1.54)	0.276	–	–
PTPN2 rs7234029				
WT	Reference		–	–
HETE/HOMO	1.98 (0.60–6.49)	0.259	–	–
HLA-C rs10484554				
WT	Reference		–	–
HETE/HOMO	1.62 (0.54–4.87)	0.394	–	–
IL23R rs11209026				
WT	Reference		–	–
HETE/HOMO	1.22 (0.24–6.30)	0.813	–	–
IL12B rs6887695				
WT	Reference		Reference	
HETE/HOMO	3.06 (1.20–7.82)	0.019	4.77 (1.60–14.23)	0.005

**Table 4:** Patients’ characteristics and genotype distributions of selected SNPs according to ustekinumab discontinuation (completers vs. discontinuers).

Variable	Completers	Discontinuers	*p*-Value
	82	16	
Age [median, Q1–Q3]	55 (41–66)	44 (35–63)	0.535
Gender (M)	39 (47.6)	7 (43.8)	1.000
Disease			
CD	63 (76.8)	7 (43.8)	0.013
UC	19 (23.2)	9 (56.3)	
Baseline disease activity			
HBI [median, Q1–Q3]	4 (3–7)	8 (3–10)	0.278
pMS [median, Q1–Q3]	3 (1–6)	6 (5–7)	0.046
Baseline steroid therapy	22 (26.8)	5 (31.3)	0.763
PTPN2 rs7234029			
A/A	59 (72.0)	15 (93.8)	0.108
A/G	23 (28.0)	1 (6.3)	
G/G	-	-	
HLA-C rs10484554			
C/C	59 (72.0)	13 (81.3)	0.600
C/T	19 (23.2)	2 (12.5)	
T/T	4 (4.9)	1 (6.3)	
IL23R rs11209026			
G/G	75 (91.5)	14 (87.5)	0.183
G/A	7 (8.5)	1 (6.3)	
A/A	-	1 (6.3)	
IL12B rs6887695			
G/G	30 (36.6)	9 (56.3)	0.408
G/C	34 (41.5)	5 (31.3)	
C/C	18 (22.0)	2 (12.5)	

**Table 5:** Univariable and multivariable Cox proportional hazards regression analyses of factors associated with ustekinumab discontinuation during the 12-month follow-up.

Variable	Univariable HR (95% CI)	*p*-Value	Multivariable HR (95% CI)	*p*-Value
Age	0.99 (0.96–1.02)	0.614	–	–
Gender (M)	0.84 (0.31–2.25)	0.725	–	–
Disease				
CD	Reference		Reference	
UC	3.67 (1.36–9.86)	0.010	3.81 (1.26–11.55)	0.018
Baseline disease activity				
Remission	Reference		Reference	
Mild activity	1.07 (0.18–6.42)	0.938	0.93 (0.15–5.74)	0.937
Moderate/severe activity	5.12 (1.43–18.36)	0.012	4.66 (1.20–18.09)	0.026
Baseline steroid therapy	1.24 (0.43–3.56)	0.694	0.55 (0.16–1.84)	0.330
PTPN2 rs7234029				
WT	Reference		–	–
HETE/HOMO	0.19 (0.03–1.44)	0.109	–	–
HLA-C rs10484554				
WT	Reference		–	–
HETE/HOMO	0.64 (0.18–2.24)	0.484	–	–
IL23R rs11209026				
WT	Reference		–	–
HETE/HOMO	1.49 (0.34–6.56)	0.597	–	–
IL12B rs6887695				
WT	Reference		Reference	
HETE/HOMO	0.47 (0.17–1.26)	0.132	0.29 (0.10–0.85)	0.024

## Data Availability

The raw data supporting the conclusions of this article will be made available by the authors on request.

## Supplementary Materials

The following supporting information can be downloaded at https://www.mdpi.com/article/10.3390/ph19091428/s1, Figure S1: Reasons for ustekinumab discontinuation during follow-up.

## Abbreviations

AIC, Akaike information criterion; CD, Crohn’s disease; CI, confidence interval; EDTA, ethylenediaminetetraacetic acid; HBI, Harvey–Bradshaw Index; HETE, heterozygous variant genotype; HLA, human leukocyte antigen; HLA-C, human leukocyte antigen C; HOMO, homozygous variant genotype; HR, hazard ratio; HWE, Hardy–Weinberg equilibrium; IBD, inflammatory bowel disease; IgG1, immunoglobulin G1; IL, interleukin; IQR, interquartile range; MHC, major histocompatibility complex; OR, odds ratio; PASI, Psoriasis Area and Severity Index; PCR, polymerase chain reaction; pMS, partial Mayo Score; SFR, steroid-free remission; SNP, single-nucleotide polymorphism; TDM, therapeutic drug monitoring; TE, Tris–EDTA; Th1, T helper 1; Th17, T helper 17; TNF, tumor necrosis factor; UC, ulcerative colitis; WT, wild type.
