# Supplementary material for: Towards Precision Medicine in Inflammatory Bowel Diseases: Pharmacogenetic Predictors of Ustekinumab Effectiveness and Persistence
Source: Pharmaceuticals (Basel). 2026 Sep 10;19(9):1428. doi: 10.3390/ph19091428 (PMC13610517; doi:10.3390/ph19091428)
Supplement: Supplementary file 1 [file pharmaceuticals-19-01428-s001.zip › Figure S1 and original dataset/Supplemental materials final.pdf]

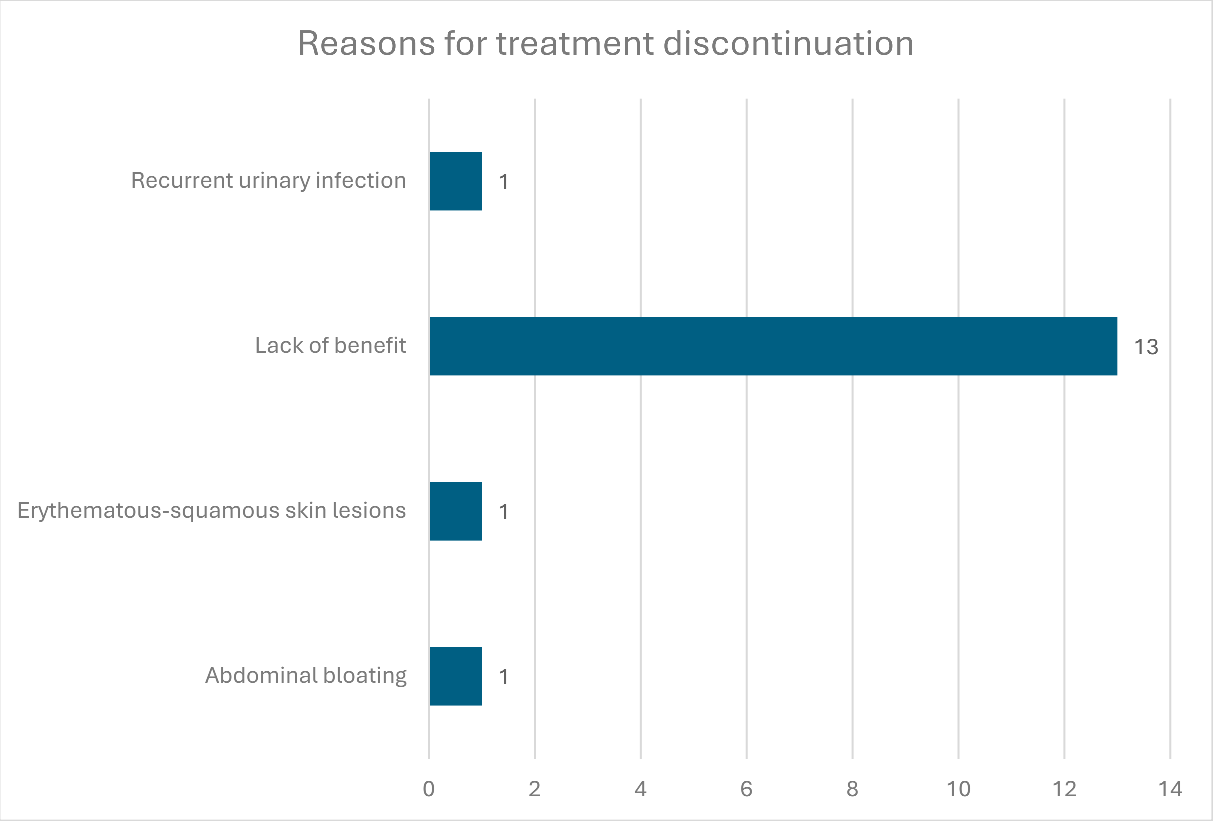

Supplementary Figure S1. Reasons for ustekinumab discontinuation during follow-up.
